# The Rediscovery of a Long Described Species Reveals Additional Complexity in Speciation Patterns of Poeciliid Fishes in Sulfide Springs

**DOI:** 10.1371/journal.pone.0071069

**Published:** 2013-08-16

**Authors:** Maura Palacios, Lenin Arias-Rodriguez, Martin Plath, Constanze Eifert, Hannes Lerp, Anton Lamboj, Gary Voelker, Michael Tobler

**Affiliations:** 1 Department of Wildlife and Fisheries Sciences, Texas A&M University, College Station, Texas, United States of America; 2 División Académica de Ciencias Biológicas, Universidad Juárez Autónoma de Tabasco, Villahermosa, Tabasco, México; 3 Evolutionary Ecology Group, J. W. Goethe University Frankfurt am Main, Frankfurt, Hessen, Germany; 4 Department for Integrative Zoology, University of Vienna, Vienna, Austria; 5 Department of Zoology, Oklahoma State University, Stillwater, Oklahoma, United States of America; University of Massachusetts, United States of America

## Abstract

The process of ecological speciation drives the evolution of locally adapted and reproductively isolated populations in response to divergent natural selection. In Southern Mexico, several lineages of the freshwater fish species of the genus *Poecilia* have independently colonized toxic, hydrogen sulfide-rich springs. Even though ecological speciation processes are increasingly well understood in this system, aligning the taxonomy of these fish with evolutionary processes has lagged behind. While some sulfide spring populations are classified as ecotypes of *Poecilia mexicana*, others, like *P. sulphuraria*, have been described as highly endemic species. Our study particularly focused on elucidating the taxonomy of the long described sulfide spring endemic, *Poecilia thermalis* Steindachner 1863, and investigates if similar evolutionary patterns of phenotypic trait divergence and reproductive isolation are present as observed in other sulfidic species of *Poecilia*. We applied a geometric morphometric approach to assess body shape similarity to other sulfidic and non-sulfidic fish of the genus *Poecilia*. We also conducted phylogenetic and population genetic analyses to establish the phylogenetic relationships of *P. thermalis* and used a population genetic approach to determine levels of gene flow among *Poecilia* from sulfidic and non-sulfidic sites. Our results indicate that *P. thermalis*' body shape has evolved in convergence with other sulfide spring populations in the genus. Phylogenetic analyses placed *P. thermalis* as most closely related to one population of *P. sulphuraria*, and population genetic analyses demonstrated that *P. thermalis* is genetically isolated from both *P. mexicana* ecotypes and *P. sulphuraria*. Based on these findings, we make taxonomic recommendations for *P. thermalis*. Overall, our study verifies the role of hydrogen sulfide as a main factor shaping convergent, phenotypic evolution and the emergence of reproductive isolation between *Poecilia* populations residing in adjacent sulfidic and non-sulfidic environments.

## Introduction

Divergent natural selection, often mediated by environmental variation, is a key driver of phenotypic evolution. Its effects can lead to the emergence of locally adapted populations that exhibit unique traits and occupy habitats with distinct combinations of environmental factors [1]. Depending on the strength of selection and rates of gene flow, such local adaptation may also cause the emergence of reproductive isolating barriers among diverging populations as a byproduct, a process known as ecological speciation [2]–[4]. Such speciation driven by adaptation has been documented in a variety of organisms and in response to a diversity of selective forces (e.g., resource exploitation [5], [6], habitat use [7], [8], and predation [9], [10]). Current research efforts focus on elucidating the range of conditions under which local adaptation is likely to translate into reproductive isolation, particularly because speciation does not appear to be an inevitable consequence of divergent selection even when phenotypic differentiation is pronounced [11]–[13]. The fact that phenotypic divergence is not necessarily tied to the emergence of reproductive isolation, as well as the dynamic nature of species boundaries, pose a challenge for efforts in biological systematics and taxonomy, which attempt to describe and organize biological diversity [14].

Historically, taxonomists catalogued species solely based on morphological trait variation (i.e., they applied a morphological species concept) without considering the underlying mechanisms contributing to phenotypic variation and speciation. Darwin [15] himself conceded that the recognition of species was often left to the opinion, experience, and expertise of naturalists. Confounding evolutionary processes and taxonomy has led to much confusion for both taxonomists and evolutionary biologists [16]. While the Modern Synthesis consolidated the definition of a biological species to being based on reproductive isolation [17], [18], a wide variety of other species concepts is currently being applied and newly developed depending on the objective at hand [19]. Consequently, there is a perpetual struggle to align the works of earlier biologists with a more modern understanding of phenotypic evolution and speciation [19], particularly in diverse and taxonomically difficult groups with a wealth of available species names, many of which currently are considered junior synonyms of older epithets [20]. Reexamination of long described taxa is critical to accurately assess biodiversity and align taxonomy with evolutionary processes leading to diversity, because phenotypic variation due to developmental plasticity [21], genetically based intraspecific polymorphisms [22], and large-scale geographic trait variation are not necessarily tied to reproductive isolation.

The genus *Poecilia*, which is part of the livebearer family Poeciliidae, represents an excellent example of the difficulties in aligning evolutionary processes and taxonomy. *Poecilia* is a diverse group of freshwater fish species that are distributed from the southeastern United States and Middle America to parts of South America and the Greater Antilles [23]. Taxonomic confusion is particularly prevalent in the *P. sphenops* (short fin molly) species group, which occurs from northern Mexico to Venezuela [24]. On one hand, these fish show tremendous variability in phenotypic traits, which has lead to the description of numerous species (many of which are currently considered synonyms) [25]. Despite clear phylogenetic structuring [26] and a long list of available names [25], species designation often remains unclear. This is predominantly caused by pronounced eco-morphological and geographic variation [27], intra-specific trait variability that appears to regularly exceed the inter-specific differences [23], [28], [29], uncertainty about whether and how morphological differences are tied to reproductive isolation [30], [31], potential hybridization and introgression among lineages [26], [32], [33], and sometimes unclear type localities of available names [25]. On the other hand, there is some well documented cases of phenotypically divergent and reproductively isolated species; yet these lineages often do not appear to be phylogenetically distinct, presumably because divergence occurred relatively recently [34]. Therefore, evaluation of species in this group in an evolutionary context is crucial for the resolution of taxonomy and for a better understanding of the historical and current processes shaping the species' evolution.

**Figure 1:**
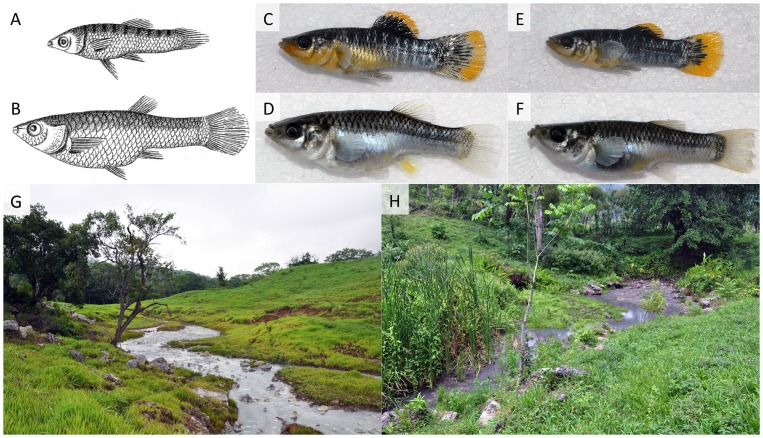
*Poecilia thermalis* and its natural habitat. A.-F. Representative specimens of *Poecilia thermalis* (males on top, females below). A. & B. represent artistic depictions of the species from the original description by Steindachner [35]. C.-F. are photos of freshly collected specimens of the *P. thermalis* from the large La Esperanza spring in 2012. G. The type locality of *P. thermalis* at La Esperanza (large spring), Chiapas, Mexico, a typical sulfidic spring habitat. H. A second, smaller sulfide spring (La Esperanza small spring) in close proximity to the type locality, which is also inhabited by *P. thermalis.*

Here, we attempt to clarify the status of one such long described species, *Poecilia thermalis* Steindachner 1863 (Fig. 1), which has had a vivid taxonomic history (Table 1). The species was originally described based on specimens that C. B. Heller collected in a sulfidic spring (La Esperanza) located in the Ixtapangajoya river drainage of Chiapas, Mexico, in 1848 [35]. The species description particularly emphasized the large head size in the available specimens. Shortly after the species description, Günther [36] thought to recognize *P. thermalis* in samples from El Salvador, but this has been largely viewed as a misidentification [37]. Subsequently, the species was subjected to various nomenclatural re-assignments by taxonomists (i.e., it has predominantly been viewed as a synonym to *P. sphenops*; see Table 1). Interestingly, all prior nomenclatural acts revolving around this species have either been based solely on the consultation of the misidentified specimens [23], [38], [39] or on information given in the species description (without actually examining specimens [24], [37]). Because the species has not been collected since 1848, we revisited the type locality of *P. thermalis* based on descriptions in Heller's [40] autobiographical account of his travels in southern Mexico to investigate the status of this enigmatic species. Our interest was fueled by the sulfidic nature of *P. thermalis*' habitat, which was emphasized both in the species description and Heller's field accounts [35], [40]. In drainages adjacent to the Río Ixtapangajoya (namely the Tacotalpa and Puyacatengo drainages to the east, and the Pichucalco drainage to the west), evolutionarily independent *Poecilia* lineages have colonized springs with high concentrations of toxic hydrogen sulfide (H_2_S) [34]. These sulfide spring inhabitants are phenotypically distinct from the closely related *P. mexicana* in non-sulfidic environments within the same drainage and are characterized by morphological, physiological, behavioral, and life history adaptations that show strong signals of convergent evolution across drainages [34]. In particular, sulfide spring fishes are characterized by enlarged heads and correlated increases in gill surface area, which facilitates oxygen acquisition in hypoxic sulfide spring environments and directly affects survival [34], [41], as well as physiological and biochemical adaptations that reduce the impacts of sulfide toxicity [42]. In conjunction with adaptive trait divergence, the sulfide spring populations are reproductively isolated from adjacent populations from non-sulfidic waters despite small geographic distances (in some instances <100 meters) and a lack of physical barriers that would prevent fish migration [43], [44]. Reproductive isolation appears to be mediated particularly by natural and sexual selection against immigrants [43]. The taxonomic status of sulfide spring populations varies across drainages; sulfide spring residents in the Tacotalpa and Puyacatengo drainages are considered ecotypes of the widespread *P. mexicana* despite clear morphological differences and strong reproductive isolation, while sulfide spring residents in the Pichucalco drainage have been described as a distinct species, *Poecilia sulphuraria* [45].

**Table 1:** Summary of the taxonomic history of *Poecilia thermalis* and *P. sulphuraria* in chronological order.

Author	Year	Action taken	Species examined	Location of examined specimens
Steindachner	1863	Original description of *P. thermalis*	*P. thermalis*	La Esperanza, Chiapas, Mexico
Günther	1866	Misidentified *P. thermalis*	*Poecilia cf. salvatoris*	Thermal Springs, El Salvador
Regan	1907	Revised misidentified *P. thermalis* and synonymized it with *P. salvatoris*	*Poecilia cf. salvatoris*	Thermal Springs, El Salvador
Rosen & Bailey	1963	Synonymized *P. thermalis* with *P. sphenops*	*Poecilia cf. salvatoris*	Thermal Springs, El Salvador
Alvarez	1947	Original description of *P. sulphuraria*	*Poecilia sulphuraria*	Baños del Azufre, Tabasco, Mexico
Miller	1983	Synonymized *P. thermalis* with *P. mexicana*	*Poecilia cf. salvatoris*	Thermal Springs, El Salvador
Poeser	2003	Revalidated *P. thermalis*	None	
Poeser	2011	Validated *P. thermalis*	None	

The table provides information about the investigating scientist, the taxonomic modification made, the species investigated, and the origin of investigated specimens.

Consequently, this study investigated the re-discovered sulfide spring species *Poecilia thermalis* to examine whether it shows similar evolutionary patterns (i.e., phenotypic trait divergence and reproductive isolation) as other sulfidic populations in the region and to shed light on its taxonomy. Specifically, we used morphological, phylogenetic, and population genetic approaches to address three major questions: (1) How do specimens from the type locality of *Poecilia thermalis* phenotypically compare to other *Poecilia* populations from sulfidic and non-sulfidic spring habitats in the region? Using a geometric morphometric approach, we tested for potential morphological convergence between *P. thermalis* and other sulfide spring fish in southern Mexico. We also explored the similarity of body shape between historical samples of *P. thermalis* [35] to current populations of sulfidic and non-sulfidic spring fish, including recently collected *P. thermalis* from the type locality. (2) What is the phylogenetic relationship of *P. thermalis* to other mollies? Based on mitochondrial and nuclear markers from a broad taxonomic sampling of *Poecilia,* we elucidated the phylogenetic position of *P. thermalis*. We were particularly interested in determining whether the species represents a unique sulfide-adapted lineage within *Poecilia*. (3) Is *P. thermalis* genetically isolated from adjacent *Poecilia* populations? Sulfide spring populations of *Poecilia* consistently exhibit a high degree of reproductive isolation from non-sulfidic populations despite the small spatial distance and a lack of migratory barriers [46]–48. Using a population genetic approach based on microsatellites, we quantified gene flow between the endemic sulfide-adapted species of *P. thermalis* and *P. sulphuraria*, and *P. mexicana* populations from adjacent non-sulfidic habitats.

## Materials and Methods

### Ethics Statement

All procedures conducted for this study were approved by the Institutional Animal Care and Use Committee at Oklahoma State University (ACUP: AS10-15) and permits issued by the Municipio de Tacotalpa-Tabasco (DFET/23/2011), as well as the Mexican Federal Agencies SEMARNAT (SGPA/DGVS/04315/11 for *Poecilia sulphuraria*) and CONAPESCA (DGOPA.09004.041111.3088 for *Poecilia sp.*). Details about the selection of focal populations are given below for each section separately.

### Study sites and sample collection

**Figure 2:**
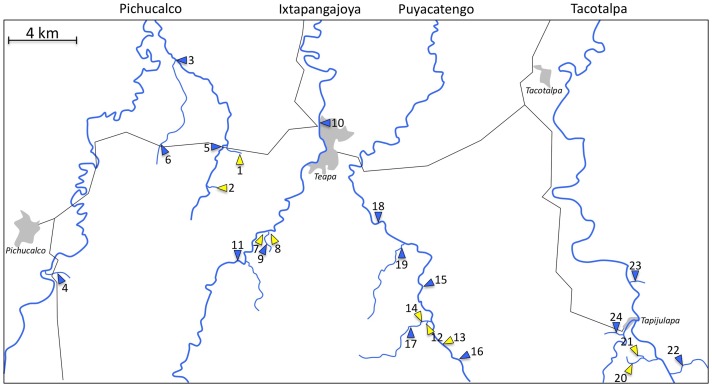
Overview of the study area and sampling localities of three *Poecilia* species in southern Mexico. Sampling of *P. thermalis*, *Poecilia mexicana mexicana*, and *P. sulphuraria* in southern Mexico. The colors represent sulfidic (yellow) and non-sulfidic (blue) sites and the numbers represent localities as described in Table 2 of the main manuscript. For orientation purposes, we included black lines representing major roads and gray areas representing major towns in the region.

Our study area lies in the foothills of the Sierra Madre de Chiapas in the northeastern part of the state of Chiapas, where the mountains meet the wide floodplains of the state of Tabasco. Here, four tributaries of the Río Grijalva, the Tacotalpa, Puyacatengo, Ixtapangajoya, and Pichucalco (from east to west), provide a system of naturally replicated non-sulfidic and adjacent sulfidic habitats (see Figure 2). The presence of high concentrations of hydrogen sulfide (H_2_S) in the sulfidic springs is a result of a nearby, active volcano, El Chinchón, and bacterial activity [49]–[51]. Besides the presence of H_2_S, the sulfidic spring environments differ from non-sulfidic habitats in a variety of environmental factors, including reduced oxygen concentration, structural habitat differences, reduced species richness, and reduced photoautotrophic primary production [34], [52], [53]. In all cases, sulfidic habitats drain directly into non-sulfidic habitats in the same drainage, and there are no major physical barriers preventing movement of fish, especially during high flow periods. The distances between sulfide spring habitats to the closest freshwater habitat range from <50 m to about 500 m.

While abiotic environmental parameters of other sites investigated here have been published in detail before [34], the type locality of *P. thermalis* at ‘La Esperanza’ has not been resampled since Heller's expedition. Exploration of the general area in May and June of 2012 revealed not one, but two proximate sulfide springs, a larger one (site 7) and a smaller one (site 8) separated by a freshwater tributary (site 9) of the Río Ixtapangajoya (Fig. 2). Based on descriptions in his autobiography, it is evident that Heller collected the type specimens from the larger spring (hence this larger spring should be considered as the type locality), which represents a cluster of relatively high discharge sulfide springs that forms a distinct tributary flowing over gravelly bottom for about 200 meters before merging with the Río Ixtapangajoya over a small drop. In this habitat, we measured the following water parameters (*N* = 4 for all measurements): sulfide concentration: 216±38 μM; dissolved oxygen: 1.31±0.39 mg/l; pH: 6.9±0.0; specific conductivity: 4.132±0.083 mS/cm; temperature: 28.7±0.9°C. The smaller spring represents a group of lower discharge springs in a swampy area with dense reeds. The site is merely a shallow pool (about 15×20 m) with a narrow outflow that – at least during our visit at the end of the dry season – eventually disappeared into pasture grounds (i.e., there was no direct, permanent connection to the Río Ixtapangajoya less than 150 m away). Here, we measured the following water parameters (*N* = 4 for all measurements): sulfide concentration: 41±3 μM; dissolved oxygen: 1.67±0.21 mg/l; pH, 7.0±0.1; specific conductivity: 2.979±0.071 mS/cm; temperature: 27.2±0.3°C. Overall, the physiochemical conditions in the two La Esperanza springs aligned well with data collected over multiple years in sulfide springs of other drainages [34].

All specimens for this study were collected using seines, euthanized with buffered MS222 immediately after capture, and fixed in a 10% formaldehyde solution for geometric morphometric analyses. All specimens are housed in the Department of Zoology, Oklahoma State University. In addition, we took fin clips (right pectoral fin) that were preserved in 95% ethanol and stored at 4°C for molecular analyses.

### Geometric morphometrics

The specimens sampled for the geometric morphometric analyses broadly covered the study region, including all currently known sulfide springs inhabited by *Poecilia* species. Our sampling included the southern subspecies of *P. mexicana mexicana* from a variety of non-sulfidic habitats in all drainages, sulfidic ecotypes of *P. m. mexicana* in the Tacotalpa and Puyacatengo drainages as well as the previously described sulfide spring endemics *P. thermalis* (Ixtapangajoya drainage) and *P. sulphuraria* (Pichucalco drainage; see Table 2 for an overview). We analyzed a total of 1099 specimens from the four drainages for a total of 23 localities.

**Table 2:** Overview of samples used in this study for morphometric and population genetic analyses.

No	Site	H_2_S	Latitude, longitude	Species	*N* body shape lateral (males/females)	*N* body shape dorsal (males/females)	*N* population genetics
**Río Pichucalco drainage**							
1	Baños del Azufre	+	17.552, −92.999	*P. sulphuraria*	54/54	52/53	25
2	La Gloria	+	17.532, −93.015	*P. sulphuraria*	29/28	28/30	24
3	Río Pichucalco	−	17.605, −93.036	*P. mexicana*	22/30	23/30	
4	Arroyo Rosita	−	17.485, −93.104	*P. mexicana*	27/29	27/28	25
5	Río El Azufre, west branch	−	17.556, −93.008	*P. mexicana*	36/53	46/47	55
6	Arroyo Raphael	−	17.558, −93.043	*P. mexicana*			16
**Río Ixtapangajoya drainage**							
7	La Esperanza, large spring	+	17.511, −92.983	*P. thermalis*	24/30	24/30	36
8	La Esperanza, small spring	+	17.511, −92.980	*P. thermalis*	31/29	30/30	24
9	Tributary to Río Ixtapangajoya	−	17.510, −92.980	*P. mexicana*	3/26	2/26	18
10	Río Teapao	−	17.555, −92.952	*P. mexicana*	6/11	6/11	25
11	Río Ixtapangajoya	−	17.495, −92.998	*P. mexicana*	39/32	44/32	24
**Río Puyacatengo drainage**							
12	La Lluvia, small spring	+	17.464, −92.895	*P. mexicana*	40/25	40/25	
13	Puyacatengo springs	+	17.458, −92.889	*P. mexicana*	13/4	13/4	
14	La Lluvia, big springs	+	17.464, −92.896	*P. mexicana*	5/11	4/10	
15	Río Puyacatengo road crossing	−	17.470, −92.896	*P. mexicana*	8/7	8/8	
16	Río Puyacatengo upstream	−	17.456, −92.888	*P. mexicana*	2/11	2/11	
17	La Lluvia upstream	−	17.461, −92.897	*P. mexicana*	5/14	5/15	
18	Río Puyacatengo at Vincente Guerrero	−	17.510, −92.914	*P. mexicana*	31/45	31/45	
19	Tributary to Río Puyacatengo	−	17.504, −92.909	*P. mexicana*	12/24	12/24	
**Río Tacotalpa drainage**							
20	El Azufre II	+	17.438, −92.775	*P. mexicana*	11/30	10/30	
21	El Azufre I	+	17.442, −92.775	*P. mexicana*	47/47	46/47	
22	Arroyo Bonita	−	17.427, −92.752	*P. mexicana*	14/24	12/25	
23	Arroyo Tres	−	17.484, −92.776	*P. mexicana*	9/22	9/22	
24	Arroyo Tacubaya	−	17.454, −92.785	*P. mexicana*	15/30	15/30	

The table highlights the collection sites of *P. mexicana*, *P. thermalis*, and *P. sulphuraria* samples used for the morphological analyses and populations genetics. Also indicated are presence (+) and absence (−) of hydrogen sulfide (H_2_S), GPS coordinates, species, and the total number of specimens examined, split by sex (only for morphology).

We conducted a geometric morphometric analysis of body shape both from the lateral and dorsal view, as body shape has been shown to be a reliable indicator for convergent evolution in sulfide spring environments [34], [54]. For all specimens, lateral and dorsal photographs were taken using a Nikon D90 camera mounted on a copy stand. We digitized 16 lateral and 9 dorsal landmark points using tpsDig2 [55] (see Figure S1A, 1B in File S1 for details on landmark locations). We analyzed lateral and dorsal landmarks separately and performed a geometric morphometric analysis based on the coordinates of the digitized landmarks [56]. Landmark coordinates were aligned using least-square superimposition as implemented in the program tpsRelw [57] to remove effects of translation, rotation, and scale. Based on the aligned coordinates, we calculated centroid size and partial warp scores with uniform components (weight matrix) for each individual. Unless otherwise stated, all statistical analyses were performed using SPSS 20 (IBM Inc.). All raw data used for the analyses described below are archived on http://datadryad.org under the DOI number associated with this publication.

The weight matrices obtained from the geometric morphometric analyses were first subjected to principal components analyses (PCA) using a covariance matrix to reduce data dimensionality. We retained 9 PC axes with an eigenvalue greater than 1 for the lateral dataset (explaining >95% of variation) and 8 PC axes for the dorsal dataset (explaining >96% of variation). Individual PC axis scores were used as dependent variables in multivariate analyses of covariance (MANCOVA). Assumptions of multivariate normal error and homogeneity of variances and co-variances were met for all analyses performed. *F*-values were approximated using Wilks' lambda and effect strengths by use of partial eta squared (*η*_p_^2^). We also calculated the relative variance as the partial variance for a given term divided by the maximum partial variance value in a model [58]. We included sex, habitat type (H_2_S present or not), drainage, and site (nested within the H_2_S × drainage interaction) as well as all interaction terms as independent variables. Centroid size was included in the models as a covariate to control for multivariate allometry.

Since random nested factors are not applicable for MANCOVAs, and the use of fixed effects can inflate type I error rates when nested terms are significant, we also analyzed shape variation using a mixed-model nested analysis of covariance (ANCOVA) [59]. To do so, we calculated divergence scores for each individual along the sulfide/non-sulfide gradient based on a divergence vector as defined by Langerhans [59]. Individual divergence scores were used as dependent variables in ANCOVAs with the same model structure as outlined above, except that site was designated a random factor to account for the fact that only a random subset of sites where *Poecilia* occurs was analyzed for this study. Shape variation along the first two PC axes and along the sulfide/non-sulfide divergence axes was visualized with thin-plate spline transformation grids using tpsRegr [60].

To fully examine the multidimensional affinities of different *Poecilia* populations relative to each other, including information from both the lateral and the dorsal projection, weight matrices for both projections were combined and subjected to a principal components analysis, from which we retained 14 axes with an Eigenvalue >1. Population-specific estimated marginal means for each axis were calculated using a MANCOVA model as detailed above and used to create a dissimilarity matrix that was subjected to a hierarchical cluster analysis using the neighbor-joining algorithm [61].

Finally, we tested whether the type specimens of *P. thermalis* collected in 1848 by Heller clustered with specimens we collected from the two La Esperanza springs in 2012. To do so, lateral photographs were taken from the available syntypes in the collection of the Natural History Museum in Vienna (*N* = 18), and we digitized the same lateral landmarks as for all other specimens. We compared the museum specimens to the samples obtained from the two La Esperanza sulfide springs, the two most proximate non-sulfidic locations in the same drainage (tributary to Río Ixtapangajoya and Río Ixtapangajoya proper), and – given the phylogenetic affinity of *P. thermalis* to *P. sulphuraria* (see below) – to the specimens obtained from the Baños del Azufre and La Gloria sulfide springs. Landmark coordinates from said collections were aligned separately. The weight matrix was then subjected to PCA, and the effects of sex and allometry removed from the dataset by using the residuals of a preparatory MANCOVA, in which the principal component scores were used as dependent variables, centroid size as a covariate, and sex as an independent variable. We then conducted a discriminant function analysis (DFA) to elucidate whether museum specimens were classified to the Esperanza sulfide springs based on body shape data. We used a cross-validation technique where discriminant functions were generated based on the data of contemporary samples (training data), and classification probabilities of museum specimens (testing data) to any of the six populations were calculated based on the established functions.

### Phylogenetic analyses

To establish the phylogenetic relationships of *P. thermalis*, we sequenced a set of genes in specimens from select non-sulfidic and sulfidic habitats included in the morphometric analyses (Table S1 in File S1). In addition, we broadened our taxon sampling by adding several other species of the subgenus *Mollienesia* (including the endemic sulfide spring species *P. sulphuraria*, the Southern and Northern Mexican subspecies *P. m. mexicana* and *P. m. limantouri*, as well as *P. butleri*, *P. sphenops, P. latipinna,* and *P. caucana*) and more distant groups in the genus *Poecilia* (sensu lato; including, *Limia vittata*, *L. dominicensis*, *L. melanogaster*, *Acanthophacelus reticulata*, *A. wingei*, *Micropoecilia bifurca*, *M. parae*, *Pamphorichthys hollandi*, and *P. minor*). The distantly related species *Cnesterodon decemmaculatus* and *C. hypselurus* were used as outgroups to root phylogenetic trees. A complete list of all taxa examined, along with locality information and GenBank Accession numbers, is provided in Supplementary Table S1 in File S1.

The total genomic DNA was extracted from ethanol-preserved fin clips with the DNeasy Blood & Tissue Kit (Qiagen, Inc., Valencia, CA) following the manufacturer's protocol. The samples were amplified for several presumably neutral genes commonly used for phylogenetic reconstruction in fishes. Focal genes included the mitochondrial cytochrome *b* gene (cyt *b*, 1,140 base pairs) with LA and HA primers [62], the mitochondrial gene NADH subunit 2 (ND2, 1,047 bp) with ND2B-L [63] and ASN [64] primers. The nuclear genes amplified included exon 3 of recombination activating gene-1 (Rag1, 1,561 bp), a portion of the 7 trans-membrane receptor region of Rhodopsin (Rh, 822 bp), and exon 1 of myosin heavy polypeptide 6 (myh6, 767 bp) with the primers and protocol following previously published PCR protocols [65], [66]. PCR products were purified with Exosap-IT enzyme reaction (GE Healthcare Bio-Sciences Corp., Piscataway, NT), directly sequenced with a dye-labeled terminator kit (Big Dye Terminator version 3.1, Applied Biosystems, Foster City, CA), and run on an ABI automated sequencer (Applied Biosystems, Foster City, CA). Sequence electrophenograms were edited with Sequencher version 4.8 (Gene Codes) and aligned with MAFFT v. 6.0 [67].

We tested for incongruence between mitochondrial (mtDNA) and nuclear (nDNA) markers to determine evidence of introgression. Given the agreement between both datasets (data not shown), we used a concatenated dataset for further analyses. We used MrModeltest version 2.3 [68] to determine the most likely model of DNA substitution among 24 candidate models on a fixed BioNJ-JC tree based on the Akaike information criterion (AIC) (Table S2 in File S1). We also compared likelihood scores between Bayes runs of 12,000,000 generations of mtDNA as a single unit, partitioned by gene and by position to determine the most informative partition. The best likelihood score was observed in the codon partition dataset and used for analyses where possible.

For maximum likelihood (ML) analyses, we used RAxML GUI version 1.0 [69], [70] run to conduct 500 Rapid Bootstrap searches followed by an ML search. We ran the complex general time reversible (GTR) + Γ (Gamma distribution for rate variation among sites) model because RAxML does not implement simpler models. We also used GARLI version 2.0 [71] to perform ML bootstrap searches (500 replicates) on the concatenated datasets under the corresponding best model selected (Table S2 in File S1). The bootstrap trees were summarized with a Sumtrees script with a 50% percent majority rule consensus parameter in DendroPy 3.10.1 [72].

Bayesian analyses were run twice independently in MrBayes version 3.2.1 [73], [74], under models of nucleotide substitution uniquely defined for the partition of each data set (Table S2 in File S1) implementing two runs with four chains under default parameters. Appropriate “burn-in” (i.e., samples discarded prior to reaching a stationary posterior distribution) was determined based on small and stable average standard deviation of the split frequencies, potential scale reduction factor close to 1 (see MrBayes manual), and stable posterior probability values examined in Tracer version 1.5 [75]. Pairwise genetic distances based on the concatenated dataset were calculated under the Kimura-2 parameter in MEGA version 5 [76] with pairwise deletion for missing data.

### Population genetics

Besides the two populations of *P. thermalis*, samples for population genetic analyses included two proximate non-sulfidic sites within the Ixtapangajoya drainage to test for gene flow between adjacent populations from sulfidic and non-sulfidic waters. Given the phylogenetic clustering of *P. thermalis* with *P. sulphuraria* from the Pichucalco drainage (see below), we also included both known *P. sulphuraria* populations as well as adjacent *P. mexicana* samples from that drainage into our analyses (Table 2). We used 17 previously developed microsatellite markers [77], [78] to genotype a total of 272 samples and arranged the microsatellites into three multiplex reactions [43]. Data from 80 specimens (Baños del Azufre and Puente El Azufre II) were re-analyzed from a previous study [43]. All raw data used for the population genetic analyses are archived on http://datadryad.org under the DOI number associated with this publication.

We extracted DNA using the NucleoSpin®Tissue kit (Macherey-Nagel). Microsatellites were amplified with the Type-it Microsatellite PCR kit from Qiagen (Hilden, Germany). The PCR protocol included an initial denaturation step for 5∶00 min at 95°C, 30 cycles of 1∶30 min at 60°C, and 0∶30 min at 72°C, followed by a final extension step for 30∶00 min at 60°C. The 5 µl reaction mix included 2.5 µl Type-it master mix, 0.4 µl primer mix, 0.4 µl Q-solution, 0.9 µl RNase-free water, and 0.8 µl template DNA. PCR products were analyzed on a CEQ2000 sequencer (Beckman) Coulter; denaturation at 90°C for 2 min, injection at 2.0 kV for 30 s, separation at 6.0 kV for 45 min) along with the manufacturer's internal size standard. Samples were screened using Genome Lab GeTX 10.2 software (Beckman Coulter) and alleles were called manually.

We employed the software STRUCTURE 2.3.4 [79] to identify the number of genetically distinct clusters (K) and then used the method of Evanno et al. [80] and the web-based software STRUCTURE HARVESTER 0.6.93 [81] to detect the uppermost level of population differentiation. In addition, we calculated pairwise *F*_ST_-values between all population pairs and conducted a Principal Component Analysis (PCA) to further examine genetic distinctiveness between populations using GenAlEx 6.5 [82].

## Results

### Phenotypic variation

**Figure 3:**
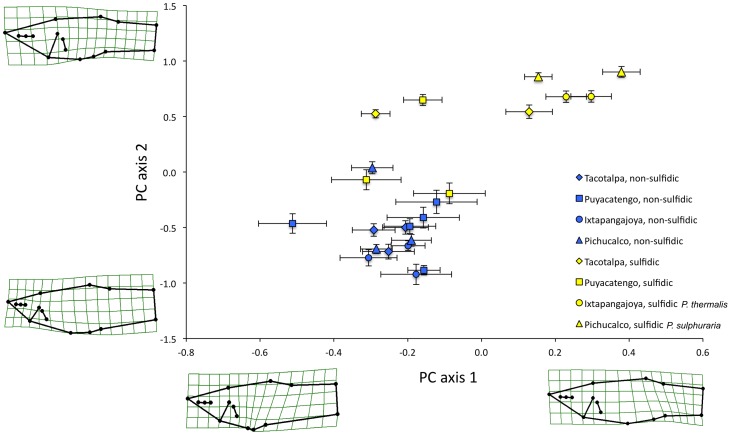
Body shape variation of *Poecilia* species in the lateral projection. Depicted are mean principal component scores along the first two principal component axes for each site for *P. thermalis* (yellow circles), *P. sulphuraria* (yellow triangles), as well as sulfidic and non-sulfidic populations of *P. mexicana* across the 23 study sites in southern Mexico. The thin-plate spline transformation grids represent shape variation along each principal component axis.

For the lateral projection (*N* = 1099 individuals), body shape varied in the position of the anal fin along PC axis 1 and the head size along PC axis 2 (Fig. 3). ‘Sex’ and the ‘presence of H_2_S’ explained the majority of body shape variation in our dataset (Table 3). ‘Sex’ particularly accounted for variation along the first PC axis (males have a more anterior anal fin position, as this fin is modified into a copulatory organ, the gonopodium). ‘Presence of H_2_S’ explained variation along the second PC axis (with sulfide spring fish having larger heads than fish from non-sulfidic habitats). All other factors and the interaction terms also had significant effects on body shape, but only ‘centroid size’, ‘drainage’, ‘site’, and the interaction of ‘H_2_S × drainage’ explained an appreciable amount of variation (relative variance >0.1; Table 3).

**Table 3:** Results of a multivariate analysis of covariance on lateral body shape of *Poecilia* from sulfidic and non-sulfidic habitats.

Effect	*F*	Hypothesis *df*	Error *df*	*P*	Partial Eta Squared	Relative variance
Intercept	143.786	9.0	1059.0	<0.001	0.550	0.672
Centroid size	148.242	9.0	1059.0	<0.001	0.557	0.680
**Sex**	**531.555**	**9.0**	**1059.0**	**<0.001**	**0.819**	**1.000**
**H_2_S**	**301.533**	**9.0**	**1059.0**	**<0.001**	**0.719**	**0.878**
Drainage	65.127	27.0	3093.5	<0.001	0.355	0.433
Site (H_2_S × Drainage)	12.579	135.0	8254.7	<0.001	0.149	0.182
Sex × H_2_S	7.581	9.0	1059.0	<0.001	0.061	0.074
Sex × Drainage	4.703	27.0	3093.5	<0.001	0.038	0.046
H_2_S × Drainage	46.531	27.0	3093.5	<0.001	0.282	0.344
Sex × H_2_S × Drainage	2.843	27.0	3093.5	<0.001	0.024	0.029

Effects explaining a relative variance ≥0.8 are highlighted in bold font.

**Figure 4:**
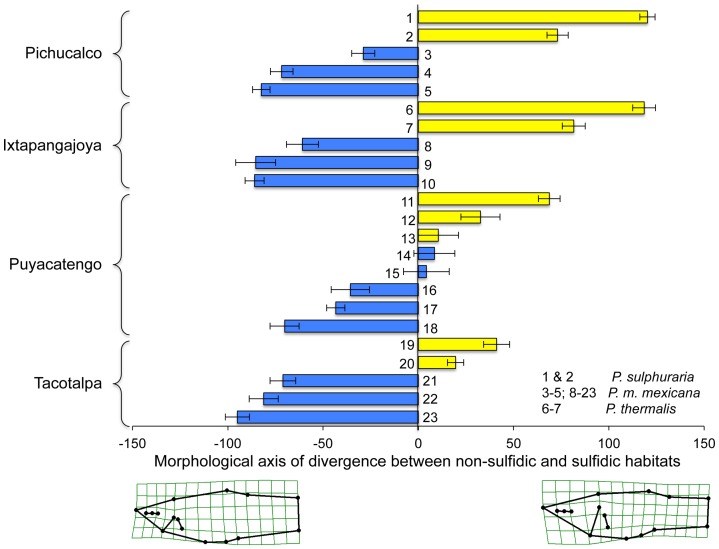
Convergent changes in body shape of *Poecilia* species from sulfidic and non-sulfidic habitats in the lateral projection. Depicted are the mean divergence scores (± SEM; derived from the H_2_S term in the MANCOVA) for each site for the three formal species (*P. thermalis*, *P. sulphuraria*, and *P. mexicana*) across the 23 sites in southern Mexico from sulfidic (yellow) and non-sulfidic (blue) populations including. The numbers correspond to sites as described in Table 2.

These general patterns, and particularly the strong differentiation between ecotypes from sulfidic and non-sulfidic springs, were confirmed in the analysis of divergence vector scores with ‘site’ being treated as a random factor (Table 4). Visualization of shape variation along the sulfide-non-sulfide divergence vector corroborated head size as the primary difference between ecotypes (Fig. 4). Inspection of divergence scores indicated that *P. thermalis*, just like *Poecilia* from the Tacotalpa and the Pichucalco drainages, exhibits a body shape typical for sulfide spring populations. In the Puyacatengo drainage, differentiation between sulfidic and non-sulfidic ecotypes was less clear-cut, with some populations exhibiting more intermediate body shapes (Fig. 4).

**Table 4:** Results of a univariate analysis of covariance on the lateral body shape divergence vector scores between *Poecilia* from sulfidic and non-sulfidic habitats.

Effect	*F*	Hypothesis *df*	Error *df*	*P*	Partial Eta Squared	Relative variance
Intercept	0.530	1.0	93.3	0.468	0.006	0.007
Centroid size	0.547	1.0	1067.0	0.460	0.001	0.001
Sex	90.425	1.0	1067.0	<0.001	0.078	0.088
**H_2_S**	**123.415**	**1.0**	**16.2**	**<0.001**	**0.884**	**1.000**
Drainage	3.092	3.0	15.1	0.059	0.381	0.431
Site (H_2_S × Drainage)	15.411	15.0	1067.0	<0.001	0.178	0.201
Sex × H_2_S	33.671	1.0	1067.0	<0.001	0.031	0.035
Sex × Drainage	0.232	3.0	1067.0	0.874	0.001	0.001
H_2_S × Drainage	4.092	3.0	15.0	0.026	0.450	0.509
Sex × H_2_S × Drainage	0.247	3.0	1067.0	0.863	0.001	0.001

Effects explaining a relative variance ≥0.8 are highlighted in bold font.

**Figure 5:**
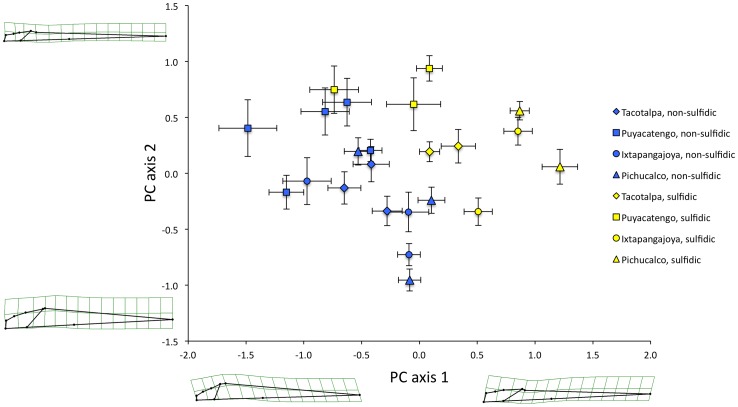
Body shape variation of *Poecilia* species in the dorsal projection. Depicted are mean principal component scores along the first two principal component axes for each site for *P. thermalis* (yellow circles), *P. sulphuraria* (yellow triangles), as well as sulfidic and non-sulfidic populations of *P. mexicana* across the 23 study sites in southern Mexico. The thin-plate spline transformation grids represent shape variation along each principal component axis.

For the dorsal projection (*N* = 1093), analyses revealed that body shape particularly varied in head length and width, mouth width, and body width at the insertion of the pelvic fins (see Fig. 5). ‘Presence of H_2_S’ explained most variation in body shape, with ecotypes from sulfidic and non-sulfidic habitats particularly segregating along the first PC axis. As for the lateral projection, all other factors and the interaction terms were also significant predictors of body shape, but only ‘sex’, ‘drainage’, ‘site’, and the interaction of ‘H_2_S × drainage’ had relative variance >0.1 (Table 5).

**Table 5:** Results of a multivariate analysis of covariance on dorsal body shape of *Poecilia* from sulfidic and non-sulfidic habitats.

Effect	*F*	Hypothesis *df*	Error *df*	*P*	Partial Eta Squared	Relative variance
Intercept	6.221	8.0	1039.0	<0.001	0.046	0.086
Centroid size	6.154	8.0	1039.0	<0.001	0.045	0.084
Sex	15.988	8.0	1039.0	<0.001	0.110	0.206
**H_2_S**	**148.306**	**8.0**	**1039.0**	**<0.001**	**0.533**	**1.000**
Drainage	33.562	24.0	3014.0	<0.001	0.205	0.385
Site (H_2_S × Drainage)	6.632	120.0	7409.6	<0.001	0.087	0.163
Sex × H_2_S	2.605	8.0	1039.0	0.008	0.020	0.038
Sex × Drainage	3.191	24.0	3014.0	<0.001	0.024	0.045
H_2_S × Drainage	17.269	24.0	3014.0	<0.001	0.117	0.220
Sex × H_2_S × Drainage	3.105	24.0	3014.0	<0.001	0.023	0.043

Effects explaining a relative variance ≥0.8 are highlighted in bold font.

**Figure 6:**
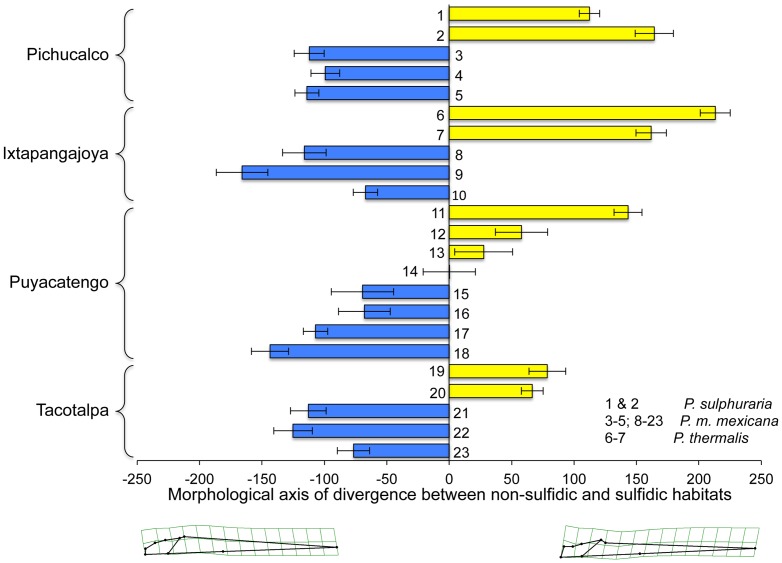
Convergent changes in body shape of *Poecilia* species from sulfidic and non-sulfidic habitats in the dorsal projection. Depicted are mean divergence scores (± SEM; derived from the H_2_S term in the MANCOVA) for each site for the three formal species (*P. thermalis*, *P. sulphuraria*, and *P. mexicana*) across the 23 sites in southern Mexico from sulfidic (yellow) and non-sulfidic (blue) populations. Numbers correspond to sites as described in Table 2.

The strong differentiation between ecotypes in dorsal body shape was confirmed in the analysis of divergence vector scores with site being treated as a random factor (Table 6). Visualization of shape variation along the sulfide-non-sulfide divergence vector indicated that sulfide spring fish had longer heads, wider mouths, but narrower bodies (Fig. 6). Differentiation between ecotypes from sulfidic and non-sulfidic springs was highly significant for all sites and drainages investigated, including both *P. thermalis* populations. The only exception was site 14 (Río Puyacatengo road crossing), which exhibited an intermediate morphology (Fig. 6).

**Table 6:** Results of a univariate analysss of covariance on the dorsal body shape divergence vector scores between *Poecilia* from sulfidic and non-sulfidic habitats.

Effect	*F*	Hypothesis *df*	Error *df*	*P*	Partial Eta Squared	Relative variance
Intercept	17.959	1.0	311.3	<0.001	0.055	0.060
Centroid size	18.768	1.0	1046.0	<0.001	0.018	0.020
Sex	16.682	1.0	1046.0	<0.001	0.016	0.018
**H_2_S**	**167.011**	**1.0**	**15.9**	**<0.001**	**0.913**	**1.000**
Drainage	1.469	3.0	15.0	0.263	0.227	0.249
Site (H_2_S × Drainage)	7.703	15.0	1046.0	<0.001	0.099	0.108
Sex × H_2_S	2.391	1.0	1046.0	0.122	0.002	0.002
Sex × Drainage	0.491	3.0	1046.0	0.689	0.001	0.001
H_2_S × Drainage	3.775	3.0	15.1	0.033	0.429	0.470
Sex × H_2_S × Drainage	1.756	3.0	1046.0	0.154	0.005	0.005

Effects explaining a relative variance ≥0.8 are highlighted in bold font.

**Figure 7:**
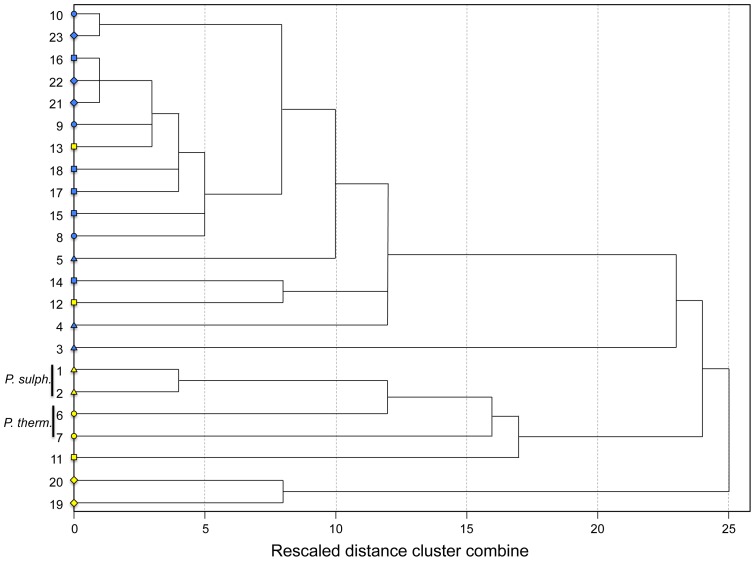
Hierarchical cluster analysis of *Poecilia* populations from sulfidic and non-sulfidic environments based on body shape variation in the lateral and dorsal projections. Colors denote sulfide-adapted (yellow) and non-adapted (blue) populations of three species, *Poecilia thermalis* (yellow circles), *P. sulphuraria* (yellow triangles), and *P. mexicana*. The shapes represent the drainages (diamonds- Tacotalpa, squares- Puyacatengo, circles- Ixtapangajoya, and triangles- Pichucalco) and the numbers correspond to sites as described in Table 2.

The cluster analysis based on the combined lateral and dorsal datasets grouped 7 of 9 populations from sulfidic habitats together in a discrete cluster, highlighting a strong convergent pattern of body shape evolution in sulfide springs (Fig. 7). The two notable exceptions were fish from the La Lluvia big spring and the Puyacatengo springs (both in the Puyacatengo drainage), which were nested within non-sulfidic populations (Fig. 7). Most importantly, however, the *P. thermalis* samples from both La Esperanza springs in the Ixtapangajoya drainage formed a cluster with the *P. sulphuraria* samples from the Baños del Azufre and the La Gloria springs in the Pichucalco drainage.

Finally, the DFA (subset of *N* = 397) clearly grouped the 18 type specimens, for which we were able to obtain lateral body shape data, with contemporary sulfide spring samples, not with proximate non-sulfidic samples. Nonetheless, only 72.2% of samples were assigned to the large La Esperanza spring (Fig. S2 in File S1; Table S3 in File S1), with the remaining individuals grouped either with *P. sulphuraria* samples from the La Gloria springs (16.7%) and Baños del Azufre (11.1%). It should be noted at this point that the sample size for historical specimens was relatively low, such that within population variation in body shape may be underestimated. The examination of additional syntypes, which were not available at the time of our study, consequently could lead to a lower overall classification success.

### Phylogenetic analyses

**Figure 8:**
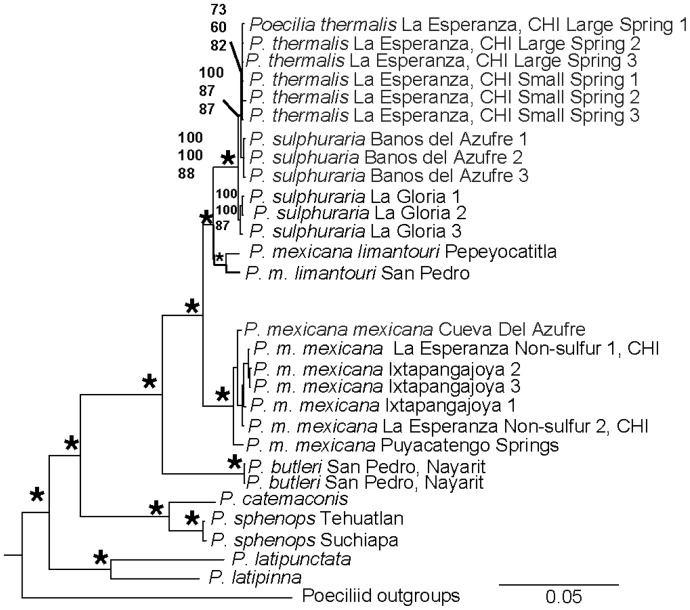
Bayesian tree from phylogenetic analysis of *Poecilia* species for five markers rooted with poeciliid outgroups. Phylogenetic analyses of two mitochondrial and three nuclear genes (5337 base pairs) yielded nodal support values (in percent) represent (from top to bottom) Bayesian Posterior Probabilities, as well as RAxML, and GARLI bootstrap support values. Asterisks denote nodal support of ≥95% for all three methods. Nodes with no values present either had low values or were of little interest for this study.

The evolutionary relationships in our phylogenetic analyses corroborate previously observed relationships in *Poecilia* among the main lineages of *Acanthophacelus*, *Micropoecilia*, *Limia*, *Pamphorichthys*, and *Mollienesia* [65], [66]. The results also show similar patterns previously observed in the relationships among species within the subgenus *Mollienesia*, with the sailfins, *P. latipinna* and *P. latipunctata*, forming a monophyletic group (Fig. 8; 100% BSS; 100% BPP). They are closely related to the monophyletic the shortfin group (100% BSS; 100% BSP [34]) with an average genetic divergence of 7.6% (Table S4 in File S1). Within the shortfin mollies, there is a separation among the *P. sphenops* clade (*P. catemaco* and *P. sphenops*; 100% BSS; 100% BSP) and the *P. mexicana* clade (*P. butleri*, *P. sulphuraria*, *P. thermalis*, *P m. mexicana*, *P. m. limantouri;* 100% BSS; 100% BSP) with an average genetic divergence of 6.7%. Phylogenetic analyses strongly (100% BSS and 100% BPP) indicate that *P. mexicana*, *P. sulphuraria*, and *P. thermalis* represent a monophyletic group. However, we did not find *P. sulphuraria* to be monophyletic, as *P. thermalis* is most closely related to the *P. sulphuraria* from the Baños de Azufre population, a relationship that is highly supported (87% BSS; 100% BSP). Genetic divergence between *P. thermalis* and this population of *P. sulphuraria* was generally low (0.1%) and only slightly less than the divergence to the La Gloria population (0.2%; Table S4 in File S1).

### Population genetic analyses

**Figure 9:**
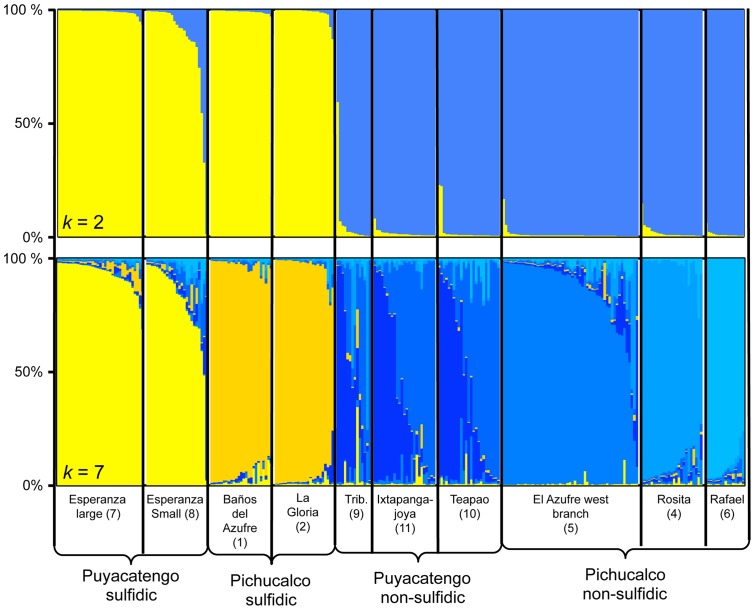
Genetic structure among populations based on microsatellites analysis in *N* = 272 individuals from 10 populations. The top panel is a bar plot showing the assignment scores of individuals by STRUCTURE with *K = 2* with yellow representing *P. thermalis* and *P. sulphuraria* as a cluster from the Puyacatengo and Pichucalco sulfidic drainages and blue representing *P. m. mexicana* from non-sulfidic sites in the same drainage. The bottom panel is a bar plot showing the assignment scores clustering at *K = 7*, the second most likely number of distinct groups.

Our Bayesian clustering analysis uncovered *K* = 2 as the uppermost hierarchical level of population structure. *Poecilia sulphuraria* (*sensu lato*; i.e, including both the Baños del Azufre and the La Gloria population) and *P. thermalis* together formed one genetic cluster that was distinct from all *P. mexicana* populations (Fig. 9). The only exception was one animal caught in the small Esperanza spring, which was assigned to *P. mexicana*, not *P. thermalis*. Another peak for ln P (X|K) – i.e., the second most likely level of population structure according to Evanno et al. [80] – was obtained for *K* = 7. In addition to detecting population genetic structure within and between drainages in *P. mexicana* a clear separation between *P. thermalis* and *P. sulphuraria* (*s.l.*) became apparent (Fig. 9), indicating low recurrent gene flow between them. Pairwise *F*_ST_-values (Table S5 in File S1) revealed significant genetic differentiation between populations of *P. thermalis* and *P. sulphuraria* (0.086–0.103) as well as between populations within each species (*P. thermalis*, 0.031; *P. sulphuraria*, 0.082). Further support for genetic differentiation between the two species was obtained from the individual-based PCA (Fig. S3 in File S1).

## Discussion

The re-discovered species, *Poecilia thermalis,* is a highly endemic sulfide spring fish found to inhabit two proximate sulfidic springs in the Ixtapagajoya drainage of southern Mexico. Morphological features exhibited by *P. thermalis* closely resemble those of other sulfide spring fishes, independent of their phylogenetic relationship, highlighting strong patterns of convergent morphological evolution in sulfidic environments. Phylogenetic analyses placed *P. thermalis* as sister taxon to one (Baños del Azufre) of the two populations of the sulfidic spring endemic *P. sulphuraria* [45] from the Pichucalco drainage with low – albeit significant – genetic distance. In addition, population genetic analyses detected little or no current gene flow between *P. thermalis* and *P. m. mexicana* from adjacent non-sulfidic habitats or *P. sulphuraria* from sulfidic springs in the Pichucalco drainage, respectively, indicating an independent evolutionary trajectory of *P. thermalis*.

### Morphological variation

In general, body shape analyses of sulfidic and non-sulfidic fishes in southern Mexico demonstrated a clear differentiation between habitat types across drainages, except for the Puyacatengo drainage. In the latter, phenotypic differentiation – particularly in the lateral projection – was less pronounced and more gradual, which could be driven by the higher spatial heterogeneity in the presence of H_2_S (see [43] for a discussion). Our results support previous analyses suggesting that fish in the Puyacatengo drainage were not as differentiated along a speciation continuum as sulfide spring fish from other drainages, because either they colonized sulfide springs more recently or gene flow across habitat types constrains phenotypic divergence [43]. Despite the strong patterns of convergence, we also found significant differences in body shape between sulfidic fish among drainages, indicating that there is both convergent and non-convergent aspects trait differentiation in response to sulfide exposure.

The phenotypic analyses of lateral body shape indicated that fishes from sulfidic and non-sulfidic habitats primarily differ in head size, with populations from sulfidic habitats having significantly larger heads. As such, our study validated previous findings from the genus *Poecilia* [34] and other poeciliids [54] with the most comprehensive sampling of sulfide spring mollies to date. Previous studies have shown that head size is positively correlated gill surface area, which is adaptive in sulfidic environments because sulfide springs exhibit low oxygen concentrations and sulfide detoxification requires additional oxygen [34]. Modification of respiratory morphological traits in conjunction with changes in respiratory behavior represent a critical adaptation mediating survival in the sulfidic and hypoxic environments [41], [83].

Our study is the first to examine body shape variation in the dorsal projection and found sulfide spring fishes to exhibit longer and wider heads, wider mouths, and narrower bodies. Sulfidic spring fishes are known to rely on aquatic surface respiration (ASR), i.e., they skim the water from the air-water interface (with higher dissolved oxygen concentrations) using their gills [41], [83]. Wider heads and mouths likely are adaptive, because they maximize the uptake of surface water as reported in neotropical characids [84] and serrasalmids [85], [86], which exhibit temporary dermal swellings of the lower jaw allowing for an increased efficiency of ASR when exposed to hypoxic conditions. The decrease in body width may be associated with a reduced body condition previously documented in fish from sulfidic habitats [52], [87], [88].

Our morphological analyses also provided critical insights about the rediscovered *P. thermalis*. Most importantly, specimens collected by Heller in 1848 mostly grouped with samples from our 2012 survey, suggesting we have visited the locality described in Heller's autobiography [40]. We found *P. thermalis* to exhibit a typical sulfide spring body shape and to be phenotypically similar to *P. sulphuraria*. Qualitatively, this is also the case for color patterns (pronounced turquoise highlights on the abdomen with a relatively dark dorsal coloration, which are not found in sulfidic *P. mexicana* populations) and lateral lip appendages on the lower jaw (authors, personal observation), which are mentioned in the species description of *P. sulphuraria* [45]. Note, however, that such lip appendages are not a diagnostic trait for *P. sulphuraria (s. l.)*, as specimens from the La Gloria population do not exhibit this morphological trait [89]. Despite the close morphological affinity of *P. thermalis* to *P. sulphuraria* (as compared to sulfidic and non-sulfidic populations of *P. mexicana*), our analyses indicated significant differences in body shape between the two species both in the lateral and dorsal projection.

### Phylogenetic analyses and population genetics

The broad phylogenic relationships uncovered in our study match the patterns of other studies [26], [34], [65], [66]. We found *P. thermalis* collected in both La Esperanza springs (Ixtapangajoya drainage) to be sister to *P. sulphuraria* from the Baños del Azufre population (Pichucalco drainage), which together were sister to *P. sulphuraria* from La Gloria (also Pichucalco drainage) and formed a monophyletic group. This group (*P. thermalis* and *P. sulphuraria* together) was more closely related to the northern Mexican subspecies of *P. m. limantouri* than the southern *P. m. mexicana* populations from adjacent non-sulfidic sites, corroborating earlier investigations [34]. This suggests that colonization of sulfide springs in the Ixtapangajoya and Puyacatengo drainages by the *P. m. limatouri-*like ancestor shared by *P. thermalis* and *P. sulphuraria* occurred earlier than sulfide spring colonization by *P. m. mexicana* in the other drainages. This is reflected in higher genetic divergences between sulfide spring and adjacent non-sulfidic populations in the Ixtapangajoya and Pichucalco drainages (2.0–2.2% in mitochondrial genes) compared to the Puyacatengo and Tacotalpa drainages (0.1–0.4%; see [34] for a discussion).

Our results indicate that sulfide springs in the Pichucalco and the Ixtapangajoya drainages were not colonized independently, but rather *P. sulphuraria* and *P. thermalis* are of a single evolutionary origin despite their current distribution in independent drainages, which in the area of the sulfide springs are separated by mountainous terrain reaching more than 500 meters above the surrounding elevation. This can be explained by the dynamic nature of the courses of major river systems in southern Mexico [90], [91]. Historically, the Grijalva River was an independent deltaic system that followed the course of Ixtapangajoya river [92], presenting an opportunity for connections between currently independent tributaries, particularly during periods of heavy rain and flooding between tributaries. However, considering the reduced viability of sulfide adapted fish in non-sulfidic environments [43], [93] and consequently the absence of sulfide-adapted ecotypes even in proximate freshwater habitats [44], it remains unclear how colonization through stretches of unsuitable habitats was possible even in the presence of potential connections among drainages. Hence, the alternative hypothesis is that colonization of different springs in the two drainages could have occurred independently by a once widespread ancestor (a lineage with close affinities to extant northern Mexican *P. m. limantouri*) with standing genetic variation for traits adaptive to sulfidic springs. Such a scenario was recently supported in stickleback, where low rates of gene flow from freshwater to marine populations maintain freshwater alleles in the marine environments at low frequency, such that selection upon colonization of a new freshwater system can rapidly reassemble freshwater ecotypes based on allelic variants already present in the ancestral population [94], [95]. The currently available data does not allow for rigorously testing these contrasting hypotheses, and additional research including a more thorough analysis of the northern Mexican *P. m. limantouri* is required to elucidate historical patterns of sulfide spring colonization in the Ixtapangajoya and Puyacatengo drainages. Nonetheless, our data indicate that sulfide spring colonization may not have occurred independently in different drainages, adding an additional layer of complexity in the analysis of speciation patterns in sulfidic spring fishes.

Our population genetic analyses largely supported the phylogeny in that the uppermost level of population differentiation included two clusters distinguishing between populations in sulfidic (*P. sulphuraria* and *P. thermalis*) and non-sulfidic (*P. mexicana*) environments irrespective of the drainage of origin. As such, the results generally support previously uncovered patterns of strong reproductive isolation between sulfide spring residents and fish from adjacent non-sulfidic sections of the same drainage [43]. Reproductive isolation among ecotypes is at least partially mediated by natural and sexual selection against immigrants, where migrant individuals from the opposite habitat type have reduced survivability and are discriminated against during mate choice [43], [46], [94]. Our analyses also found strong support for genetic structure with *K* = 7 divergent clusters. At this finer scale, the two *P. thermalis* populations from the Ixtapangajoya drainage were clearly distinct from the two *P. sulphuraria* populations in the adjacent Pichucalco drainage, reflecting the absence of gene flow due to the lack of contemporary connections between the two drainages.

### Taxonomic considerations and conclusions

The taxonomic history of *Poecilia thermalis* remained uncertain since its original description by Steindachner in 1863. Our study revisited the status of *P. thermalis* based on recently collected material from the type locality and museum specimens using morphological, phylogenetic, and population genetic approaches. Based on our findings, we can clearly reject the previously prevalent notion that *P. thermalis* Steindachner 1863 is synonymous to either *P. salvatoris* [38], *P. sphenops* [96], or *P. mexicana* (sensu stricto, [39]). However, in relation to the sulfide spring populations from the Pichucalco drainage, currently denominated *P. sulphuraria* Alvarez 1947, taxonomic change can proceed in the form of two alternatives. Sulfide spring populations from the Ixtapangajoya (*P. thermalis*) and the Pichucalco (*P. sulphuraria*) could be considered as derivatives from the same evolutionary lineage and therefore considered the same species. In this case, the older names takes precedence [97] and *P. sulphuraria* [45] would be designated as a junior synonym of *P. thermalis*. Alternatively, *P. thermalis* could be designated as a valid, distinct species restricted to the Ixtapangajoya drainage, which would require the name *P. sulphuraria* to be restricted to the type locality (Baños del Azufre) and the sulfide spring population at La Gloria (currently a population of *P. sulphuraria*) to be considered a distinct species awaiting formal description. This interpretation is supported by reciprocal monophyly, significant population genetic differentiation as evident from *F*_ST_ values and principal components analysis, and significant differences in body shape among all three groups. Examination of additional characters, especially meristic traits and the structure of the male copulatory organ (gonopodium), commonly used in poeciliid systematics will hopefully lead to the resolution of the taxonomic conundrum surrounding *P. thermalis*.

Regardless of the taxonomic conclusions, our study has direct implications for the conservation of the sulfide spring populations in the Ixtapangajoya and Pichucalco drainages. Currently, *P. sulphuraria* is listed as threatened and federally protected by the Mexican government [98]. In addition, the IUCN has listed the species as critically endangered because of a very limited distribution [97], and the species is threatened by deforestation, farming, recreational activities, and more recently by extensive palm oil plantations [89]. Despite these concerns, no conservation measures have been implemented to mitigate these effects [89]. Potential taxonomic changes will require according changes in the list of endangered species in Mexico. Whether all three populations will be designated as *P. thermalis* or as three distinct species in the future, they clearly represent unique evolutionary lineages with highly restricted distributions meriting separate management and a high priority for conservation [99].

Overall, this study confirms the role of hydrogen sulfide in shaping convergent, phenotypic evolution in sulfide spring fishes and causing reproductive isolation between populations residing in proximate sulfidic and non-sulfidic environments. It also illustrates how an integrative, mechanistic approach to studying phenotypic evolution and speciation can inform taxonomy.

## Supporting Information

File S1 (PDF)
