# Supplementary material for: The Rediscovery of a Long Described Species Reveals Additional Complexity in Speciation Patterns of Poeciliid Fishes in Sulfide Springs
Source: PLoS One. 2013 Aug 16;8(8):e71069. doi: 10.1371/journal.pone.0071069 (PMC3745397; doi:10.1371/journal.pone.0071069)
Supplement: File S1 — (PDF) [file pone.0071069.s001.pdf]

Supplementary information

The rediscovery of a long described species reveals additional complexity in speciation patterns of poeciliid fishes in sulfide springs

Maura Palacios, Lenin Arias Rodriguez, Martin Plath, Constanze Eifert, Hannes Lerp, Anton Lamboj, Gary Voelker & Michael Tobler

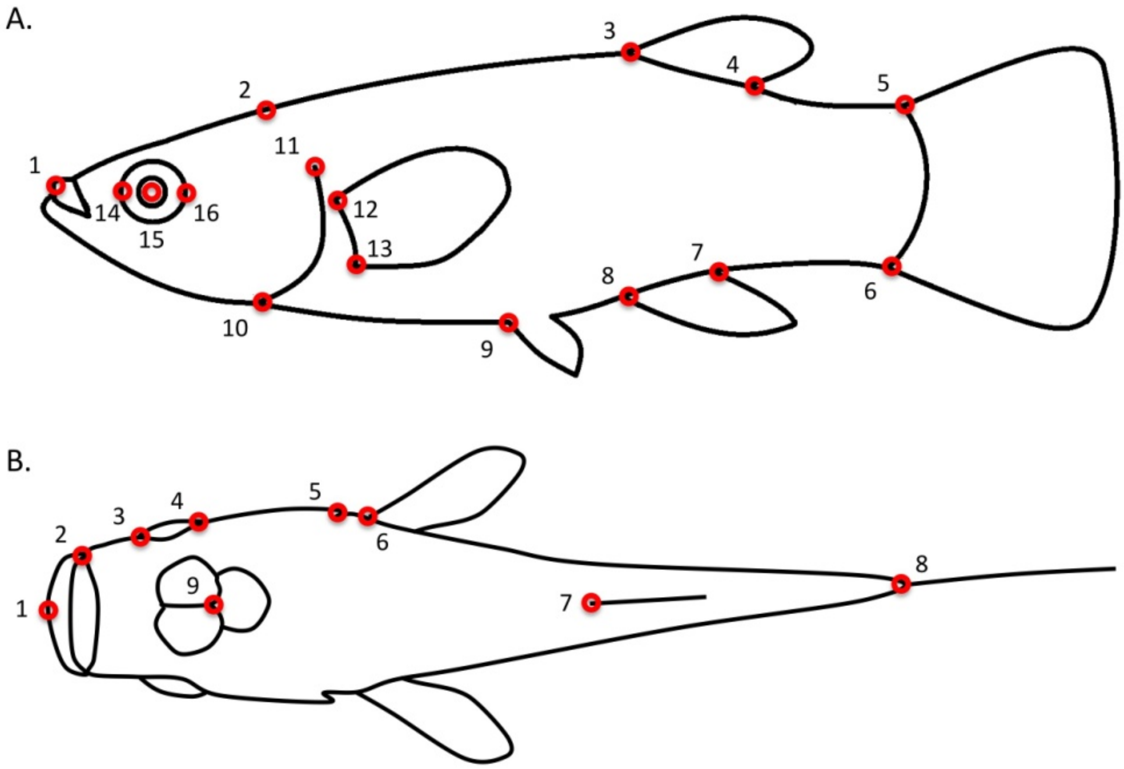

Figure S1. The 16 lateral and the 9 dorsal landmarks used for geometric morphometric analysis. A. The 16 lateral landmarks for the lateral pictures and included (1) the tip of the upper jaw, (2) the postero-dorsal corner of the head, (3) the anterior and (4) posterior insertions of the dorsal fin, (5) the dorsal and (6) ventral insertions of the caudal fin, (7) the posterior and (8) anterior junctions of the anal fin, (9) the anterior junction of the pelvic fin, (10) the bottom of the head where the operculum breaks away from the body outline, (11) the dorsal endpoint of the opercular bone, (12) the dorsal and (13) ventral insertions of the pelvic fin, as well as (14) the anterior edge, (15) the center, and the posterior edge of the eye orbit. B. The 9 dorsal landmarks included (1) center of the lower jaw, (2) the corner of the mouth where the lower and upper jaws meet, (3) the anterior and (4) posterior corners of the eye, (5) the posterior edge of the operculum, (6) the anterior insertion of the pectoral fin, (7) the anterior insertion of the dorsal fin, (8) the dorsal insertion of the caudal fin, and (9) the intersection of the last two head and the first dorsal body scales.

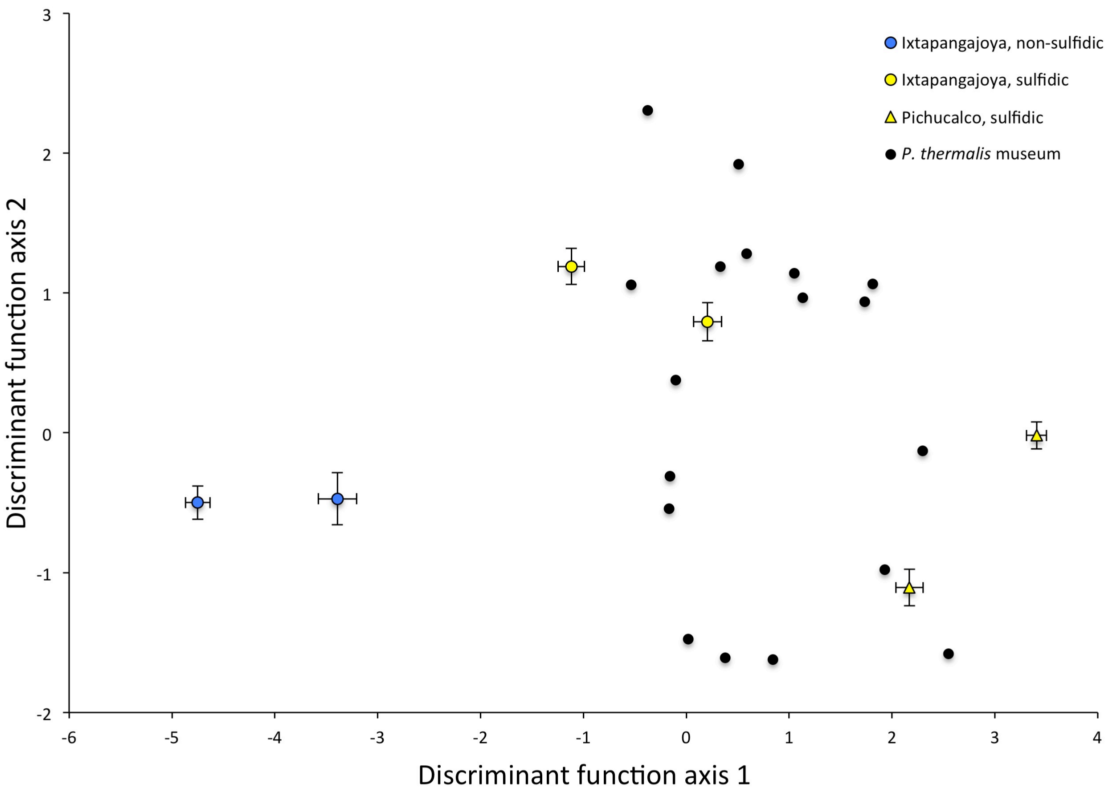

Figure S2. Results of discriminant function analysis (DFA) based on lateral body shape analysis of three *Poecilia* species. Note that historical samples of *P. thermalis* clearly cluster with contemporary samples of *P. thermalis* and *P. sulphuraria*, not with *P. m. mexicana* from adjacent non-sulfidic environments.

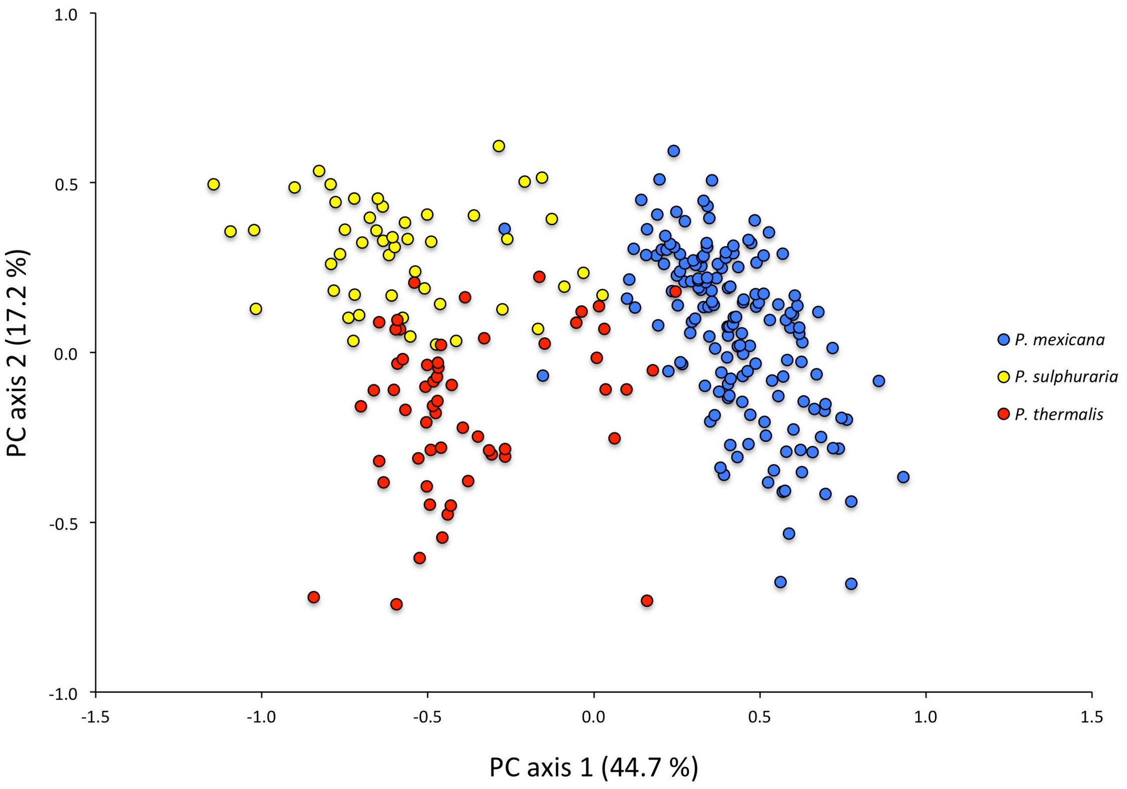

Figure S3. Principle component analysis based on the microsatellite dataset used for population genetic analyses.

Table S1. Specimens investigated for phylogenetic analyses including locality, genes, and accession numbers. Asterisks indicate samples that were sequenced specifically for the present study. For samples collected by the authors, we also included the catalog numbers of voucher specimens (MT numbers).

Taxon	Location	Drainage	State	Country	Cyt b	ND2	Myh6	RAG1	Rh
P. thermalis (MT12-30-1)	La Esperanza, large spring	Ixtapangajoya	Chiapas	Mexico	*KF276617	*KF276675	*KF276646	*KF276704	*KF276733
P. thermalis (MT12-30-2)	La Esperanza, large spring	Ixtapangajoya	Chiapas	Mexico	*KF276618	*KF276676	*KF276647	*KF276705	*KF276734
P. thermalis (MT12-30-3)	La Esperanza, large spring	Ixtapangajoya	Chiapas	Mexico	*KF276619	*KF276677	*KF276648	*KF276706	*KF276735
P. thermalis (MT12-32-1)	La Esperanza, small spring	Ixtapangajoya	Chiapas	Mexico	*KF276620	*KF276678	*KF276649	*KF276707	*KF276736
P. thermalis (MT12-32-2)	La Esperanza, small spring	Ixtapangajoya	Chiapas	Mexico	*KF276621	*KF276679	*KF276650	*KF276708	*KF276737
P. thermalis (MT12-32-3)	La Esperanza, small spring	Ixtapangajoya	Chiapas	Mexico	*KF276622	*KF276680	*KF276651	*KF276709	*KF276738
P. sulphuraria (MT12-37-1)	Baños del Azufre	Pichucalco	Tabasco	Mexico	*KF276623	*KF276681	*KF276652	*KF276710	*KF276739
P. sulphuraria (MT12-37-2)	Baños del Azufre	Pichucalco	Tabasco	Mexico	*KF276624	*KF276682	*KF276653	*KF276711	*KF276740
P. sulphuraria (MT12-37-3)	Baños del Azufre	Pichucalco	Tabasco	Mexico	*KF276625	*KF276683	*KF276654	*KF276712	*KF276741
P. sulphuraria (MT-12-36-1)	La Gloria	Pichucalco	Tabasco	Mexico	*KF276626	*KF276684	*KF276655	*KF276713	*KF276742
P. sulphuraria (MT-12-36-2)	La Gloria	Pichucalco	Tabasco	Mexico	*KF276627	*KF276685	*KF276656	*KF276714	*KF276743
P. sulphuraria (MT-12-36-3)	La Gloria	Pichucalco	Tabasco	Mexico	*KF276628	*KF276686	*KF276657	*KF276715	*KF276744
P. mexicana mexicana (MT12-31-1)	Tributary to the Rio Ixtapangajoya	Ixtapangajoya	Chiapas	Mexico	*KF276629	*KF276687	*KF276658	*KF276716	*KF276745
P. mexicana mexicana (MT12-31-2)	Tributary to the Rio Ixtapangajoya	Ixtapangajoya	Chiapas	Mexico	*KF276630	*KF276688	*KF276659	*KF276717	*KF276746
P. mex. mexicana (MT12-39-1)	Rio Ixtapangajoya	Ixtapangajoya	Chiapas	Mexico	*KF276631	*KF276689	*KF276660	*KF276718	*KF276747
P. mex. mexicana (MT12-39-2)	Rio Ixtapangajoya	Ixtapangajoya	Chiapas	Mexico	*KF276632	*KF276690	*KF276661	*KF276719	*KF276748
P. mex. mexicana (MT12-39-3)	Rio Ixtapangajoya	Ixtapangajoya	Chiapas	Mexico	*KF276633	*KF276691	*KF276662	*KF276720	*KF276749
P. mex. mexicana (MT10-35-1)	Cueva del Azufre	Tacotalpa	Tabasco	Mexico	*KF276644	*KF276673	*KF276644	*KF276702	*KF276731
P. mex. mexicana (MT09-10-1)	Puyacatengo Springs	Puyacatengo	Tabasco	Mexico	*KF276645	*KF276674	*KF276645	*KF276703	*KF276732
P. mex. limantouri (MT10-16-1)	Pepeyocatitla	Panuco	Hidalgo	Mexico	*KF276634	*KF276692	*KF276663	*KF276721	*KF276750
P. mex. limantouri (MT10-12-1)	San Pedro	Panuco	Hidalgo	Mexico	*KF276635	*KF276693	*KF276664	*KF276722	*KF276751

P. butleri (1)	San Pedro	San Pedro	Nayarit	Mexico	*KF276613	*KF276671	*KF276642	*KF276700	*KF276729
P. butleri (4)	San Pedro	San Pedro	Nayarit	Mexico	*KF276614	*KF276672	*KF276643	*KF276701	*KF276730
P. sphenops (MT10-15-1)	Tehuatlan	Panuco	Hidalgo	Mexico	*KF276611	*KF276669	*KF276640	*KF276698	*KF276727
P. sphenops (MT09-21-1)	Suchiapa	Grijalva	Chiapas	Mexico	*KF276612	*KF276670	*KF276641	*KF276699	*KF276728
P. catemaconis (MT10-23-1)	Lake Catemaco	Papaloapan	Veracruz	Mexico	*KF276610	*KF276668	*KF276639	*KF276697	*KF276726
P. latipinna	Wilmington	Cape Fear	North Carolina	U.S.	*KF276609	*KF276667	*KF276638	*KF276696	*KF276725
P. latipunctata	Ciudad Mante	Panuco	Tamaulipas	Mexico	EF017539	EF017588	EF017436	GU179287	GU179259
P. caucana	Unknown			Mexico	EF017540	EF017589	EF017437	GU179286	GU179258
Acanthophacelus reticulata	Cumaná	Litoral Caribe	Sucre	Venezuela	GU179192	GU179238	EF017434	GU179281	GU179253
A. wingei	Aquarium Stock				GU179193	GU179239	GU179267	GU179282	GU179254
Micropoecilia bifurca	Coropina Creek		Republiek district	Suriname	GU179186	GU179232	GU179261	GU179275	GU179247
M. parae	Rowa			French Guyana	GU179188	GU179234	GU179263	GU179277	GU179249
Limia dominicensis	River Picot		Pont Salomon	Haiti	EF017533	EF017582	EF017431	GU179273	GU179245
L. melanogaster	Aquarium stock				EF017534	EF017583	EF017432	GU179274	GU179246
Pamphorichthys araguaiensis	Cristalino River		Mato Grosso	Brazil	GU179195	GU179241	GU179269	GU179284	GU179256
P. minor	Máximo Lake		Amazonas	Brazil	GU179196	GU179242	GU179270	GU179285	GU179257
Cnesterodon hypselurus	Cilada		Paraná	Brazil	GU179185	GU179231	GU179260	GU179272	GU179244
C. dessemaculatus	Canãda		Artigas	Uruguay	EF017529	EF0175791	EF017427	GU179271	GU179243

Table S2. The genes partitioned by position for mitochondrial DNA and by gene for nuclear DNA for phylogenetic analyses of *Poecilia* species are described including the total length and number of parsimony informative sites in parentheses. The best-fit substitution models for each data partition for the concatenated dataset are provided as well as the likelihood score.

	**Cyt b *p. 1*** (380, 70)	**Cyt b *p. 2*** (380, 11)	**Cyt b *p. 3*** (380, 282)	**ND2 p. 1** (349, 88)	**ND2 p. 2** (349, 29)	**ND2 p. 3** (349, 252)	**Myh6** (767, 45)	**RAG1** (1561, 101)	**Rh** (822, 44)
**AIC**	SYM+I+G<br>-1455.33	HKY+I<br>-634.12	GTR+I+G<br>-4252.63	GTR+G<br>-1751.21	GTR+G<br>-967.27	GTR+G<br>-3919.00	GTR+I<br>-1695.87	GTR+G<br>-3715.77	HKY+I<br>-1729.11

Table S3. Results of the discriminant function analysis (DFA). Cross validation (i.e., assignment of museum specimens to sampling sites based on discriminant functions generated based from contemporary collections) indicated that 72.2 % of *P. thermalis* individuals were assigned to the large La Esperanza spring, 16.7 % to the La Gloria springs, and 11.1 % to the springs at the Baños del Azufre.

	**Function 1**	**Function 2**	**Function 3**	**Function 4**	**Function 5**
PC 1	0.157	-0.190	0.219	-0.052	-0.254
PC 2	1.093	0.169	0.092	0.026	-0.052
PC 3	-0.389	0.760	0.547	0.329	-0.159
PC 4	0.268	0.877	-0.098	0.222	-0.382
PC 5	0.146	0.024	0.220	0.043	0.872
PC 6	0.276	0.288	-0.410	0.375	0.261
PC 7	-0.119	-0.375	0.639	0.248	0.080
PC 8	0.172	-0.519	-0.197	0.666	0.038
PC 9	0.074	-0.299	-0.133	0.311	-0.083
Eigenvalue	9.471	0.571	0.286	0.072	0.016
% of variance	90.9	5.5	2.7	0.7	0.2
Cumulative %	90.9	96.4	99.2	99.8	100.0
Canonical correlation	0.951	0.603	0.471	0.259	0.125
Wilks' lambda	0.043	0.455	0.714	0.918	0.984
χ^2	1162.181	292.006	124.763	31.703	5.877
Df	45	32	21	12	5
P	<0.001	<0.001	<0.001	0.002	0.318

Table S4. Genetic distances (in %) for the concatenated dataset based on the mean Kimura-2 parameter model of evolution. Pair-wise comparisons are between the main lineages of *Poecilia* (asterisks indicate more than one species) and an out-group. The range within each lineage – when applicable – is shown in the diagonal.

	P. thermalis	*P. sulphuraria* (s.l.)	*P. m. limantouri*	*P. m. mexicana*	*P. butleri*	*P. sphenops**	Saifin clade*	*P. caucana*
P. thermalis	(0-0.001)							
P. sulphuraria (s.l.)	0.200	(0-0.300)						
P. mexicana limantouri	1.500	1.500	(0.700)					
P. mexicana mexicana	2.200	2.200	2.000	(0-0.200)				
P. butleri	3.900	3.900	3.800	3.800	-			
P. sphenops-clade*	6.800	6.800	6.700	6.700	6.600	(0.1-2.200)		
Sailfin-clade*	7.600	7.500	7.700	7.700	7.900	7.300	(5.200)	
P. caucana	9.600	9.600	9.200	9.300	9.300	9.400	9.300	-
Outgroup	15.60	15.600	15.40	15.30	15.40	15.100	15.10	15.00

Table S5. Pairwise F_{ST}-values calculated using GenAlEx 6.5 based on 17 microsatellite markers analyzed in 10 populations of *Poecilia* from Southern Mexico. Numbers after population names correspond to site numbers listed in Table 2 in the main manuscript. Bolded sites represent the highly endemic sulfide springs populations: Baños del Azufre and La Gloria represent populations of *P. sulphuraria* (*s. l.*) in the Pichucalco drainage and Esperanza Large and Small indicate populations of *P. thermalis* in the Ixtapangajoya drainage. The remaining populations represent *P. m. mexicana* populations from adjacent non-sulfidic habitats of both drainages. Asterisks denote significant ($P \leq 0.01$) F_{ST}-values based on randomization tests with 10^5 permutations.

	**Baños (1)**	**La Gloria (2)**	Rosita (4)	El Azufre West (5)	Rafael (6)	**Esperanza Large (7)**	**Esperanza Small (8)**	Tributary (9)	Teapao (10)
**La Gloria (2)**	**0.082***								
Rosita (4)	0.214*	0.194*							
El Azufre West (5)	0.171*	0.152*	0.084*						
Rafael (6)	0.196*	0.181*	0.062*	0.078*					
**Esperanza Large (7)**	**0.093***	**0.103***	0.214*	0.172*	0.195*				
**Esperanza Small (8)**	**0.086***	**0.092***	0.143*	0.114*	0.125*	**0.031***			
Tributary (9)	0.156*	0.143*	0.070*	0.049*	0.099*	0.188*	0.112*		
Teapao (10)	0.139*	0.133*	0.071*	0.032*	0.071*	0.137*	0.083*	0.019	
Ixtapangajoya (11)	0.154*	0.143*	0.080*	0.043*	0.095*	0.183*	0.111*	0.015	0.013
